# New patient assessment in old age psychiatry: the importance of risk assessment

**DOI:** 10.1192/pb.bp.113.046342

**Published:** 2015-10

**Authors:** Svetlana Hemsley, Rebecca McKnight, Aneeba Anwar, Sarah Jones, Lola Martos

**Affiliations:** 1 South Locality Older Adults Community Mental Health Team, Abingdon

## Abstract

**Aims and method** In recent years, the role of non-medical community mental health team (CMHT) clinicians has widened to include new patient assessments. It is unclear whether all professionals have the skills and confidence to undertake these to a high quality. This project investigated which professionals are doing new assessments, evaluated their quality and explored the assessors' unmet training needs. The study was based on the data extracted from electronic notes and a complete audit cycle in South Oxfordshire Older Adults CMHT; this was a cross-sectional study across Oxfordshire older adults services.

**Results** Most new assessments (72.4%) were done by non-medical clinicians; the majority were missing important information, especially relating to medications and risk assessment. Only 75% of assessors felt at least ‘partially confident’ to do assessments and found them stressful, with 86% keen to undertake further training.

**Clinical implications** Simple measures such as an assessment form, a programme of training seminars and adequate supervision, delivered to all CMHT clinicians, can ensure high-quality assessment in diverse clinical environments.

The discipline of psychiatry has seen many changes in practice over recent years. One major change has been the shifting roles of the multidisciplinary team to include some clinical duties traditionally undertaken by psychiatrists. New patient assessments are one task now frequently delegated to the wider multidisciplinary team.^1^ Non-medical clinicians often have capacity to see the increasing number of referrals arising from an aging population and are a more affordable option than psychiatrists.

Psychiatrists are taught assessment skills gradually throughout their training. Formal teaching in assessment occurs throughout medical school and the foundation programme; skills are then enhanced and specialised within psychiatry through weekly supervision, workplace-based assessments and postgraduate examinations. The same structure (focusing on assessment) is not necessarily in place for non-medical professionals. The revised General Medical Council (GMC) *Good Medical Practice* document provides guidance to doctors needing to delegate tasks to colleagues: ‘When delegating care you must be satisfied that the person to whom you delegate has the knowledge, skills and experience to provide the relevant care or treatment; or that the person will be adequately supervised’.^2^ Typically, non-medical clinicians are provided with a weekly team meeting in which to discuss patients with psychiatrists as well as regular individual supervision. It has been assumed that this is providing appropriate support and supervision. However, there is limited evidence pertaining to the skills and confidence that non-medical staff have in assessing newly referred patients. A report by the Royal College of Nursing has highlighted a need for continuing postgraduate education to ensure high standards of community psychiatric nursing, including updating skills as an individual's role within a team changes.^3^ Similarly, the recent Francis inquiry emphasised the importance of ensuring individuals' skills are appropriately matched to their duties to ensure global high-quality care within the National Health Service (NHS).^4^ Being asked to perform skills beyond a clinician's training can be stressful and reduce performance as well as leading to burn-out.^5^

We hypothesised that as non-medical clinicians have been asked to undertake new patient assessments, a skills gap has emerged. We designed a service evaluation to test this hypothesis. The aims were to identify which professionals are undertaking new assessments, investigate the quality of these assessments and explore the level of confidence individuals have in carrying out this work. The primary method of investigation was a complete audit cycle, complemented by a cross-sectional survey.

## Method

**Fig 1:**
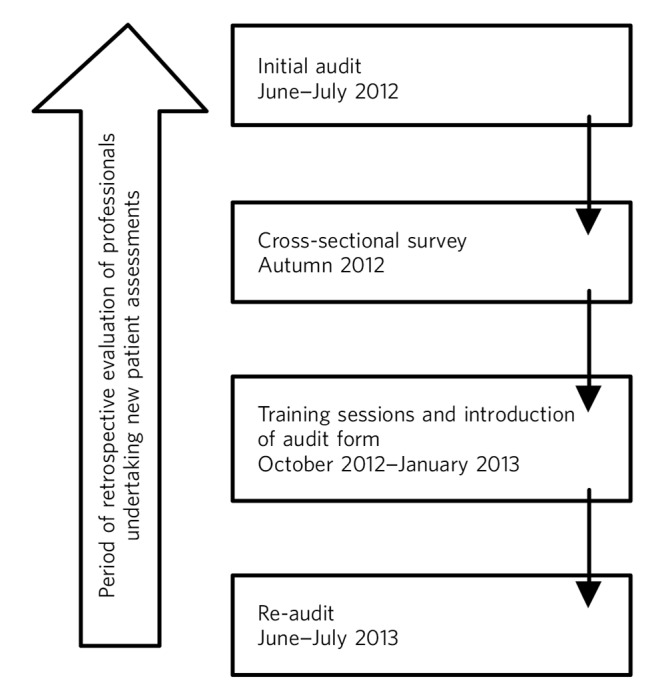
Timeline of events

This was a service evaluation undertaken in the three community mental health teams (CMHTs) of the South Locality Older Adults Service (Oxford Health NHS Foundation Trust). South Oxfordshire has a population of approximately 134 300, including 33 200 people over 60 years of age.^6^ The service evaluation was made up of three components occurring between June 2012 and July 2013 (Fig. 1). All of the components of the service evaluation were carried out in the same group of clinicians.

### Audit cycle

The aim was to determine if existing practice meets agreed standards as to what information should be recorded during a routine new patient assessment. The initial audit covered the period 1 June to 31 July 2012, re-audited for 1 June to 31 July 2013. All consecutive new referrals were included. No international or nationally agreed guidelines as to how a new patient should be assessed could be located in either 2012 or 2013, so the proxy standard used within Oxford Health NHS Foundation Trust was chosen. This includes the completion of each section of the ‘core assessment’ and ‘risk assessment’ of our RiO electronic notes system (www.servelec-group.com/Healthcare/RiO.html). Medical records were reviewed (by R.M.) first in November 2012 and then in August/September 2013: an audit tool form was completed for each individual. For each part of the assessment, if any comment was present pertaining to that area it was marked ‘yes’. This included explanations as to why information was not available at that time. During October 2012–January 2013, a pilot set of 2-h training seminars and interactive workshops covering common psychiatric presentations were delivered weekly to multidisciplinary team clinicians. A new patient assessment form was introduced at the same time (available as online supplement DS1).

### Cross-sectional survey of non-medical clinicians' training needs

We devised a 21-item questionnaire (online supplement DS2) covering confidence surrounding current assessment and education, plus unmet training need. This was distributed by email to 50 non-medical staff in 3 older-adult CMHTs. These included nursing staff, occupational therapists, social workers, mental health practitioners and psychologists. Healthcare assistants and support workers were excluded as they do not undertake new patient assessments. Participants returned an anonymous hard copy to their team manager.

### Retrospective evaluation of professionals undertaking new patient assessments

All patient contacts (new and follow-ups) covering the period 1 March 2012 to 30 April 2013 were downloaded from the RiO electronic notes system. The profession of the assessing clinician was recorded as ‘medical’ (consultants and junior doctors) or ‘non-medical’ and proportions in each category calculated.

Upon the completion of the audit cycle and survey, and using feedback from the pilot training sessions, a programme of training in assessment skills was devised. This will be delivered by psychiatrists over 7 weeks on a yearly basis to all CMHT clinicians. Staff turnover and sickness will be closely monitored.

### Statistical analysis

All results were entered into a Microsoft Excel spreadsheet for basic analysis. Audit data were analysed with SPSS v. 21 for Windows using unpaired chi-squared tests with α = 0.05.

## Results

### Audit cycle

In the initial audit, 40 consecutive referrals were received; this increased to 62 in 2013. The demographic profile of the sample remained unchanged for both audit cycles (Table 1). The professionals conducting assessments were community psychiatric nurses (CPNs; 64%), psychiatrists (20%) and occupational therapists (16%). Of the psychiatrists, there were three consultants and three psychiatric trainees.

**Table 1:** Results from audit cycle: sample characteristics

	2012	2013
Gender, male (%)	44.0	42.5

Age, mean (years)	79.5	80.0

Referral from primary care (%)	93.0	95.0

Time from assessment to documentation complete (days)	4.4	3.8

**Fig 2:**
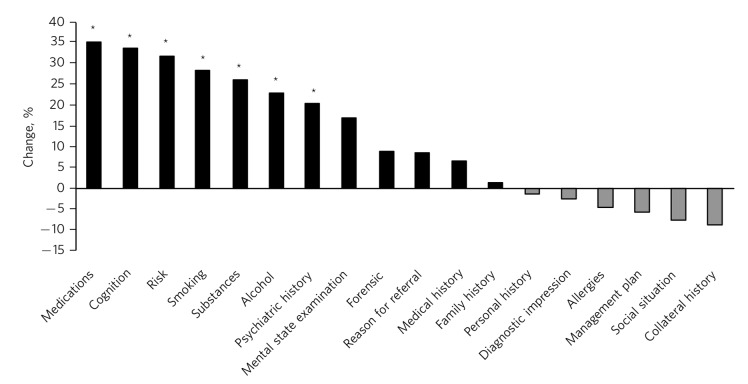
Change in contents of new patient assessments 2012-2013. *P<0.05.

In 2012, the proportion of assessments clearly marked ‘new assessment’ and properly structured with subheadings was 45%; this increased to 75% in 2013 after the introduction of an assessment form (*P* = 0.003). In 2012, the information most frequently omitted from assessment was medication history, family history, use of substances and risk assessment (Table 2). With non-psychiatrist clinicians, there was a tendency to list all living family members under family history rather than record the presence or absence of mental disorder. However, this was not the case in assessments done by psychiatrists (e.g. 2013: 83% *v.* 0%). By 2013 there had been a significant improvement in recording of psychiatric history, medications, substance use, mental state examination and risks (Fig. 2). However, out of 17 RiO subsections, only 7 had been completed in at least 75% of assessments. Psychiatrists were significantly more likely to record at least 90% of RiO sections than non-medical staff (81% *v.* 10% respectively; *P*<0.001).

**Table 2:** Results from audit cycle: assessments

	Assessments containing any information relating to the subject, %		
Subsection of RiO core assessment	2012	2013	*P*^a^
Reason for referral	82.5	90.9	

Comment on who was present at the interview	80	82	

History of presenting complaint	90	89.3	

Past medical history	52.5	59	

Past psychiatric history	52.5	72.7	0.0213

Medications	40	76	0.003

Family history	42.5	44	

Personal history	50	48.5	

Social history	92.5	85	

Alcohol	22.5	45.5	0.002

Smoking	17.5	45.5	<0.0001

Substance use	15	45.5	0.013

Forensic history	20	28.7	

Pre-morbid personality	35	28.7	

Collateral history	80	71.2	

Mental state examination	50	66.7	0.04

Risk assessment	35	66.7	<0.001

Diagnostic impression	80	77.2	

Management planning	95	89.3	

a. Chi-squared test. Non-significant *P* values not given.

Evaluation of cognition is an important part of assessment in older adults. In 2012, 70% of assessments included information on cognition and bedside cognitive tests; this fell to 58% in 2013 (*P* = 0.04). However, for patients referred with cognitive impairment, more than 95% had evidence of cognitive testing in both years.

### Cross-sectional survey of assessment confidence and unmet training needs

This survey took place between the two audits but before the pilot intervention (Table 3). Overall, 36 questionnaires were returned (72%), representing CPNs (50%), social workers (17%) and occupational therapists (17%). Three-quarters (75%) of respondents felt at least ‘partially confident’ to assess a new patient, with 22% reporting ‘no confidence’. Similarly, 75% reported feeling ‘stressed or unsupported’ while doing the assessment. Less than half of staff (44%) reported familiarity with the ICD-10 criteria for mental health disorders,^8^ and only 25% felt confident to use them to aid diagnosis. The majority of staff (80%) felt confident to ‘cluster’ patients according to type and severity of illness.

**Table 3:** Cross-sectional survey results (*n* = 36 respondents in total)

*n* = 36	Respondents %
Profession of assessing clinician	
CPN	50
Social worker	17
Occupational therapist	17
Psychologist	9
Support worker/other	8

Level of confidence in assessing a new patient	
Confident	25
Less confidence	50
No confidence	22
No comment	2.8

Familiarity with ICD-10 criteria	
Yes	44
Partly	39
No	17

Confidence in using ICD-10 criteria to make a diagnosis	
Confident	25
Less confidence	33
No confidence	28
No comment	11

How often you feel stressed, unsupported when assessing a newly referred patient?	
Most of the time	64
Sometimes	11
Not at all	22

Would you like an opportunity to undertake training in the following? (yes/no)^a^	
Information on mental disorders	75
Assessment and diagnosis of mental disorders	86
Updates from recent research	94

Six disorders clinicians would most like training on (in preference order)	
Bipolar disorder	94
Depression	83
Anxiety disorders	80
Schizophrenia	72
Personality disorder	69
Dementia	58

Preferred method of teaching (in order)	
Teaching seminars (1–2 hours)	83
Short courses (1–2 days)	77
E-learning resources	47
Formal academic course and qualification	39

How important is it to you to gain an accreditation that is recognised by your employers and other organisations for the training that you undertake?	
Very important	39
Quite important	46
Not important	13
No comment made	2

What would be the most important reason to you to undertake further training?	
To improve my clinical practice	86
For personal development	8
To enhance my CV	0
To increase the likelihood of promotion	5
Other reason	0

CPN, community psychiatric nurse.

a. Only ‘Yes’ responses given.

In all, 86% were keen for training in assessment, diagnosis and management of mental disorder. The conditions for which training was most frequently requested were (in order) bipolar disorder, depression, anxiety disorders, schizophrenia, personality disorders and dementia. The most popular methods of delivering training were seminars (83%) and 1-day short courses (78%). Most staff (85%) felt it was essential to have accreditation recognised by employers for attending training.

### Retrospective evaluation of professionals undertaking new patient assessments

Between March 2012 and April 2013, 485 new patient assessments were carried out within South Locality CMHT. In total, 41 individual clinicians were involved in the assessments, with 84% of assessments being conducted by one person. The breakdown of professionals involved was as follows: 60% CPNs, 20% psychiatrists, 16% occupational therapists, 4% social workers. The majority of new patient assessments were carried out by non-medical clinicians: 72.4% *v.* 27.6%. Similarly, 86.2% of follow-up contacts were carried out by non-medical staff. Of the new assessments by medical staff, 58% were done by consultants.

### Staff turnover and sickness

During the period from June 2012 to June 2013 the turnover of non-medical clinicians within the CMHT was 50% (compared with 12% trust wide).^8^ The average within the trust at that time was 8%. At the time of the initial audit, 12% of staff were on long-term sick leave, including two band 7 nurses (1.8% trust wide).

## Discussion

This service evaluation investigated which professionals are undertaking new patient assessments and investigated unmet training needs of the clinicians involved. We hypothesised that a skills gap has emerged as more non-medical clinicians have started to participate in assessments and that they find these new duties very stressful; our results corresponded with this hypothesis.

The Royal College of Psychiatrists recommends that CMHTs should ‘ensure the appropriate numbers of professionals with appropriate skills and competencies are in place to respond to local needs … for assessment’.^9^ Our surveys and audit clearly show that the majority of new patient assessments are now being done by non-medical clinicians and that they frequently do not feel confident to undertake this role. Not only does this pose clinical risks, but also contributes to rising financial costs due to high rates of stress-related sickness and rapid staff turnover. Our local experience is that many staff on long-term sick leave are experiencing ‘stress, anxiety or depression’; this tallies with national data.^5,8^ The way that mental health services commissioning is now linked to diagnostic clustering means that poor knowledge of diagnostic categories and grading of severity of mental health disorders could have financial implications. These implications could be reduced by providing appropriate training. Adequate knowledge of the local area and its resources is also important and this is hard to achieve with high staff turnover.

Our initial audit highlighted the poor quality of risk assessments undertaken during new patient assessments. Recent publications have alerted us to the need for high-quality risk assessment in older adults, especially for suicide and self-harm.^10^ This was an area of great concern in the 2012 audit, but the 2013 re-audit demonstrated that very simple measures – an assessment form and some pilot teaching sessions – made a significant improvement in our teams' skills and documentation. Similarly, Huh *et al*^11^ report that a 1-day course in suicide risk assessment for healthcare professionals working with older adults was highly effective at increasing staff confidence and the quality of risk assessment. The Department of Health has previously emphasised the need to provide a range of flexible approaches to education and training,^12^ and this is especially important as we increasingly recognise different styles of learning and diversify our working patterns. Key to this will be standardising access to training, for example making sure that all professionals have similar amounts of study leave provision.

We demonstrated that the majority of staff would like to undertake further training in the form of seminars or short courses, and would like accreditation for this. We have been unable to find any similar audit or research data with which to compare our results, but the Royal College of Nursing reports similar findings.^3^ Their survey of over 800 UK mental health nurses found that 89% would like further training in ‘acute mental health conditions’ and the favoured delivery methods were also teaching sessions or short courses. This work only included nurses, whereas our study includes all non-medical clinicians, but the demographics are otherwise similar. It could be argued that the ‘team’ nature of CMHTs (e.g. having staff supervision and a multidisciplinary team meeting at which new cases are presented to the consultant) allows for appropriate clinical guidance, but we have found it can be difficult to provide such guidance when faced with a lack of information gathered at an initial assessment.

### Limitations

The main limitation of this service evaluation is the sample size and the fact that it covers only one geographic area. It might also be hard to generalise to outside older adult psychiatry. The response rate for the questionnaire was low, which may be partially explained by the work having occurred during the holiday season, but other ways of reaching staff need to be investigated. It should also be remembered that staff have highly variable experience in terms of the years of practice; we cannot expect newly qualified colleagues to be comparable to those with more years of service and we did not collect this information.

## Assessment training

We propose to improve standards in new patient assessment and increase clinicians' skills and confidence in our area by providing a comprehensive training programme within normal working hours for all non-medical clinicians and junior doctors in the CMHT. This will be delivered as seven 2–3-h interactive seminars and will cover general assessment, risk assessment and management of common disorders presenting to old age psychiatry (see online supplement DS3). It will be provided at least yearly to include all incoming staff and, while led by consultants, will provide a platform for psychiatric trainees to enhance their teaching skills. Re-audits of new patient assessment structure and content will occur yearly.

Assessment is the foundation of high-quality management in psychiatry: we should work hard to ensure that all clinicians are appropriately skilled and supported to manage the vulnerable patients presenting to our services.^13^ Psychiatrists should take a leading role in delivering appropriate knowledge whereas mental health trusts should facilitate training and seek ways to encourage and reward aspiration.
